# A Web-based archive of systematic review data

**DOI:** 10.1186/2046-4053-1-15

**Published:** 2012-02-21

**Authors:** Stanley Ip, Nira Hadar, Sarah Keefe, Christopher Parkin, Ramon Iovin, Ethan M Balk, Joseph Lau

**Affiliations:** 1 Tufts Evidence-based Practice Center, Tufts Medical Center, Boston, MA 02111, USA

**Keywords:** Archive, data repository, extraction, systematic review

## Abstract

Systematic reviews have become increasingly critical to informing healthcare policy; however, they remain a time-consuming and labor-intensive activity. The extraction of data from constituent studies comprises a significant portion of this effort, an activity which is often needlessly duplicated, such as when attempting to update a previously conducted review or in reviews of overlapping topics.

In order to address these inefficiencies, and to improve the speed and quality of healthcare policy- and decision-making, we have initiated the development of the Systematic Review Data Repository, an open collaborative Web-based repository of systematic review data. As envisioned, this resource would serve as both a central archive and data extraction tool, shared among and freely accessible to organizations producing systematic reviews worldwide. A suite of easy-to-use software tools with a Web frontend would enable researchers to seamlessly search for and incorporate previously deposited data into their own reviews, as well as contribute their own.

In developing this resource, we identified a number of technical and non-technical challenges, as well as devised a number of potential solutions, including proposals for systems and software tools to assure data quality, stratify and control user access effectively and flexibly accommodate all manner of study data, as well as means by which to govern and foster adoption of this new resource.

Herein we provide an account of the rationale and development of the Systematic Review Data Repository thus far, as well as outline its future trajectory.

## Background

Tasked with effectively integrating an ever-expanding sea of scientific data, findings from systematic reviews, which combine and synthesize data from many individual studies, have become invaluable to healthcare decision-makers. In the US, the recent passage of the Patient Protection and Affordable Care Act has mandated a renewed emphasis on evidence-based practice through comparative effectiveness research. Systematic reviews are therefore poised to play an even more prominent role in US health policy. However, conducting these reviews remains time-consuming and labor-intensive [1].

A fundamental step in conducting a systematic review is identifying relevant studies and extracting the essential information from each study, information such as the study design, study population, description of the intervention and what it is being compared to and outcomes. Data across studies are then analyzed and synthesized. The extraction of one article alone may take several hours, and large reviews may include several hundred publications. Duplicate extractions by separate researchers are often conducted for verification as well, adding to resource needs and further drawing out the process. Therefore, data extraction is a major time-sink in conducting a systematic review.

As systematic reviews conducted by different organizations (serving different research purposes) often ask similar questions on the same topics, this process may be replicated numerous times for the same studies. The bodies of literature covered in such reviews frequently and necessarily overlap, and while the questions addressed vary, the data extracted from relevant publications are largely identical, representing an enormous duplication of effort. Similarly, updates of existing systematic reviews are commonly conducted to keep up with the latest scientific findings. As these updates are often conducted years after the original systematic review and often by different research groups, they frequently require re-extraction of previously reviewed studies, as data from previous incarnations of a review are not always readily available.

In June 2010, the Evidence-based Practice Center (EPC) at Tufts Medical Center, with support from the Agency for Healthcare Quality and Research (AHRQ), initiated development of the Systematic Review Data Repository (SRDR), a collaborative Web-based publicly available repository of systematic review data. As envisioned, this resource will reduce the unnecessary replication of effort in conducting a systematic review, by serving as both a central archive and data extraction tool, shared among organizations producing systematic reviews worldwide. This database will be freely accessible to facilitate evidence reviews and thus improve and speed policy-making with regards to healthcare.

## Main text

### Benefits of the Systematic Review Data Repository

The anticipated benefits of the SRDR include: a more efficient means of producing and updating systematic reviews; improved access to more detailed information by consumers of review evidence; the promotion of transparency and reliability in the systematic review process via communal review; and enhanced cooperation and utility across related resources (such as the World Health Organization's International Clinical Trials Registry Platform [2] or ClincialTrials.gov [3]). Such an archive will also facilitate novel avenues of research, such as investigations of research methodology (for example, comparing different types of outcomes across disciplines, or comparing methodological quality of studies based on funding sources), by concentrating relevant data into one readily available source. Several scenarios depicting the beneficial implications of such a repository are outlined below.

• Investigators search for data from existing systematic reviews that have been deposited in the repository. They then conduct a novel systematic review or update an existing one (or they decide to forego the effort completely) depending on the results of their search and particular research questions.

• A user misinterprets and/or mistranscribes some information during data extraction into the repository. This discrepancy is identified and flagged for correction by the community (in a manner similar to various other collaborative resources (in particular, see WikiProject Medicine [4]) and the data contributor is notified of the error.

• A stakeholder interested in a particular topic searches the repository and quickly identifies related reviews through the repository's links to other external databases.

### Types of users

To ensure that the deposited data are accurate and reliable, we plan to allow only certified users to deposit and modify data. Although data in the SRDR system will be freely accessible to anyone via the Internet, a minimal set of procedural policies to certify data depositors will be instituted. We envision a system of tiered access to SRDR data and functionality. Based on an assessment of probable use cases, our proposed system contains four types of SRDR users, each with a specific set of data access permissions. The proposed user types in order of increasing privilege are: Viewer, Commentator, Contributor and Publisher/Editor.

#### Viewer

Any interested person would be allowed to view and download published data; however, no commenting, editing or supplementation of data would be permitted. Registration prior to accessing the SRDR would be voluntary.

#### Commentator

Any interested person with permission to post comments on published SRDR entries. Such users would have to register on the SRDR website, providing basic identifying and contact information, including their name, their affiliated organization and a valid email address. Following email verification, newly registered commentators would be required to accept the terms of use for the repository's discussion features. All comments would be vetted for clarity and adherence to system rules by the SRDR support team prior to posting.

#### Contributor

A registered individual who has been given permission to contribute data to a project housed in the repository. Accounts of this type would initially be assigned via certified groups or organizations, rather than through the SRDR's Web registration. Initially, only EPCs and other invited groups would be granted rights to contribute data to the SRDR. Subsequently, additional contributors who wished to submit systematic reviews would be granted access under policies to be established with guidance from the SRDR governing body.

#### Publisher/Editor

Individuals granted the authority to publish (or make publicly viewable) a completed systematic review project on the SRDR Web site. It would be left to the participating organizations to decide who and how many members of the organization would be designated for this role.

## Discussion

### Technical challenges

To gain a thorough understanding of the issues to be considered in creating the SRDR, in June 2010 we convened a multidisciplinary panel of technical experts knowledgeable in systematic reviews and with expertise in trial registries, databases, systematic review methodologies and/or biomedical informatics. Based on the discussion during the expert panel meeting, a number of design requirements were established. First, the repository would have to be flexible and adaptable - with the ability to accommodate all manners of clinical research questions, studies and outcomes - in order to meet the needs and expectations of systematic reviewers from a variety of organizations and disciplines. Second, the repository should be easy to use and present a low hurdle for users to contribute data. Ideally, it would be readily integrated into researchers' existing review processes, facilitating more efficient systematic reviews with minimal overhead. And third, the repository should be interoperable with existing systematic review databases and resources, leveraging universal and open standards for cross-communication and collaboration.

The technical challenges in adhering to these design requirements include designing a system that balances the desired flexibility with easy-to-use editing, viewing and exporting capabilities for researchers. The amount and types of data that must necessarily be represented to accommodate the diverse needs of potential users are vast. Also, because the repository will rely largely on voluntary contribution, imposing rigid data formats or unintuitive interfaces would undermine the repository's adoption and potential value. However, such flexibility substantially complicates enabling data visualization, export (such as to statistical software) and search, in addition to adding complexity to the user-interface. While a feature-rich system is important to accommodate diverse needs, ease of use remains critical to the project's ultimate success. Overall, the goal is to permit researchers of a range of technical expertise to effectively interact with the repository, both in contributing and retrieving data.

### Non-technical challenges

We expect that the major hurdle to uptake of the SRDR will be overcoming the inertia of reviewers accustomed to their current routines, who may see little incentive to adopt new methods. In addition to providing compelling functionality that will help facilitate systematic reviews, the necessary encouragement may need to come from funders of systematic reviews, who are driven by the need for rapid production of reviews and have long-term interest in the repository's potential to drive down costs. The provision of strong and convenient technical support should also help alleviate user reluctance. The issue of custodianship, including curatorial responsibilities to assure data accuracy, will also need to be worked out. Whether the repository will eventually be a public or private, US or international entity will require careful deliberation by all involved parties.

#### Governance structure

Other, non-technical challenges that remain to be overcome primarily concern governance and data quality assurance processes. In further discussion with the expert panel, we determined that it is important to establish a governing or advisory body for this data repository. The SRDR governing body would be charged with setting overall strategic goals and priorities, and establishing and managing policies and processes, including those related to data quality control and user certification. We believe that a joint committee of various stakeholders would be best suited to guide development of the repository, by guaranteeing representation of the needs of the SRDR's various constituencies.

We believe that the structure of the proposed governing body will differ somewhat based on the eventual permanent funding source. Three possible scenarios for this source (and therefore the governing structure) are that the SRDR will continue to be fully funded and managed by the US government; that the SRDR will be funded and operated as an independent non-governmental entity, either through a stand-alone organization (such as the Patient Centered Outcomes Research Institute) or as a unit within an academic institution; or that the SRDR will operate as a commercial or fee-for-service resource. We believe the last option is undesirable because it runs counter to the SRDR's stated purpose to provide an open and freely-accessible repository to promote systematic review research. Therefore, the last scenario is not discussed further.

As previously mentioned, a governing body will oversee the management of the SRDR. Representation will likely include members from EPCs, the National Library of Medicine, the biomedical informatics community, clinical practice guideline producing entities, international stakeholders such as the Cochrane Collaboration and the Center for Reviews and Dissemination in the UK and other relevant research organizations and user communities. Should it continue to be funded by the US government, the responsible agency official would select and invite the advisory committee members following consultation with stakeholders, or oversee the contracting organization's selection process, and would determine the appropriate size of the committee and its terms of service.

Should the SRDR operate as an independent non-governmental entity, it may reside within an academic institution or exist as a standalone resource. Financial support would likely be provided in a non-commercial context, such as via public or private grants, donations and/or other similar funding mechanisms. The Cochrane collaboration might serve as an organizational model (see Cochrane Policy Manual [5]) for such an independent entity. As in the Cochrane collaboration, the SRDR's mission would be accomplished by groups of volunteers. Members of relevant stakeholder groups would come together because they share an interest in reliable, up-to-date evidence in health care.

#### Data quality control

It is essential for the SRDR's success that the deposited data are as accurate as possible. We believe that a multilayered approach to data quality control, applied both pre- and post-deposit, would best ensure that the SRDR accumulates only quality data. During data entry, we intend to implement per-field error checking (for example, built-in range checks for predefined numerical data types). Some error checking may also be user-specified (for example, range and direction for numeric data fields). In addition, process and data visualization (for example, progress meters, summary table creation and export) would assist users in entering data accurately during the data extraction process. Process and data visualization would allow project leads to review summary data in order to identify errors or missing data during the production of the systematic review.

For published data (those made publicly viewable), we propose two approaches to facilitate quality assurance: regular data audits and wiki-like editorial features for flagging or commenting on deposited data. We anticipate that the SRDR will be archiving data from a large number of systematic reviews. Thus, centralized review and curation of all deposited records will quickly become infeasible. Regular data audits of randomly selected records would strike a reasonable balance between maintaining data quality and ensuring the quality assurance workload remains feasible. These audits could initially be conducted by the Tufts EPC (as the group most familiar with the repository) and then, going forward, by each certified organization's resident SRDR coordinator. Public collaboration has been identified as potentially useful in a number of scenarios applicable to the systematic review activities sponsored by AHRQ. By enabling collaborative commentating and data review, the entire user-base can be enlisted in the quality assurance process.

#### Potential for error or misuse

Regardless of the measures outlined above, the SRDR will inevitably engender erroneous data or misuse. Such incidents may range from simple typographical errors in deposited records to more insidious mistakes of misinterpretation or misapplication of archived data. It is worth noting, however, that such errors are not restricted to the SRDR but are endemic to the creation of all systematic reviews, with the important distinction that the SRDR provides a structured mechanism for external review and correction of these errors while traditional methods generally do not.

Rather than erroneous interpretation, the most likely negative outcome to emerge from the SRDR would be abuse of the good faith in which the SRDR tools are provided. SRDR users could potentially take advantage of the repository's online extraction and analysis tools without eventually making their data publically available. It is our hope, however, that the utility the SRDR provides and the wider benefits to the research community it engenders would be obvious and compelling enough to encourage a mutually beneficial reciprocity.

## Conclusions

### Current status

As of January 2012, we have developed an initial version of the SRDR system. AHRQ plans to host the server and database for storing the data extracted into the SRDR. The current version allows contributors to initiate new systematic review projects, create extraction forms and extract data into the repository using a series of flexible interfaces. The underlying database was designed in a way such that the data, once entered into the system, will be easily retrieved and formatted into customizable summary tables for publication.

**Figure 1:**
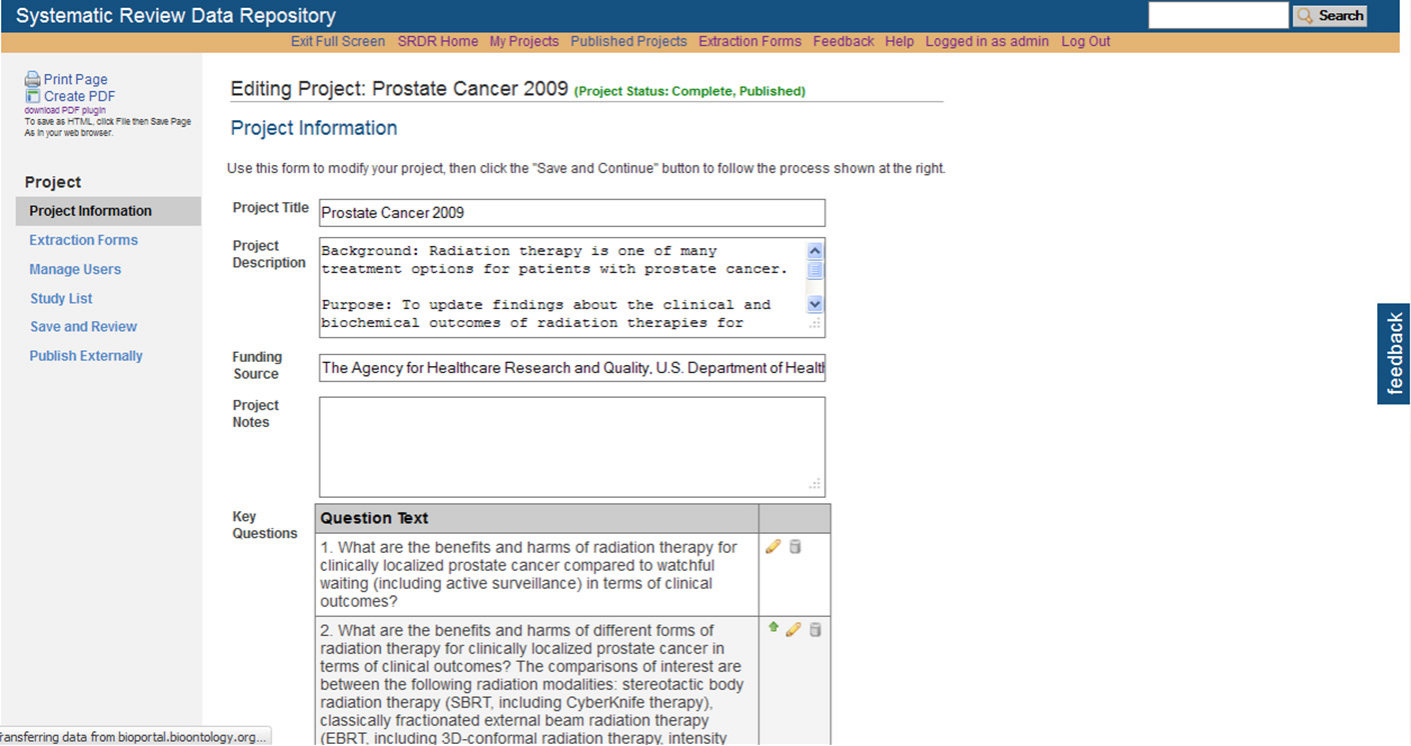
**Partial view of the Project Information Section of a Systematic Review Data Repository data extraction form**.

**Figure 2:**
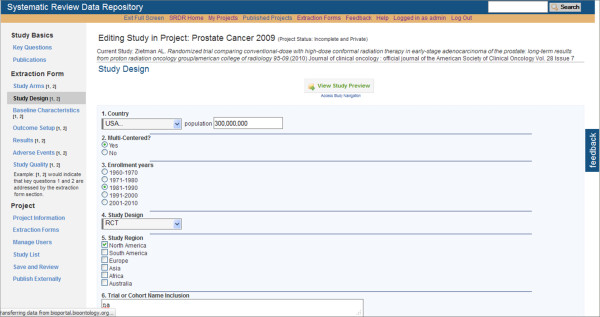
**Partial view of the Study Enrollment and Design Information Entry Section**.

**Figure 3:**
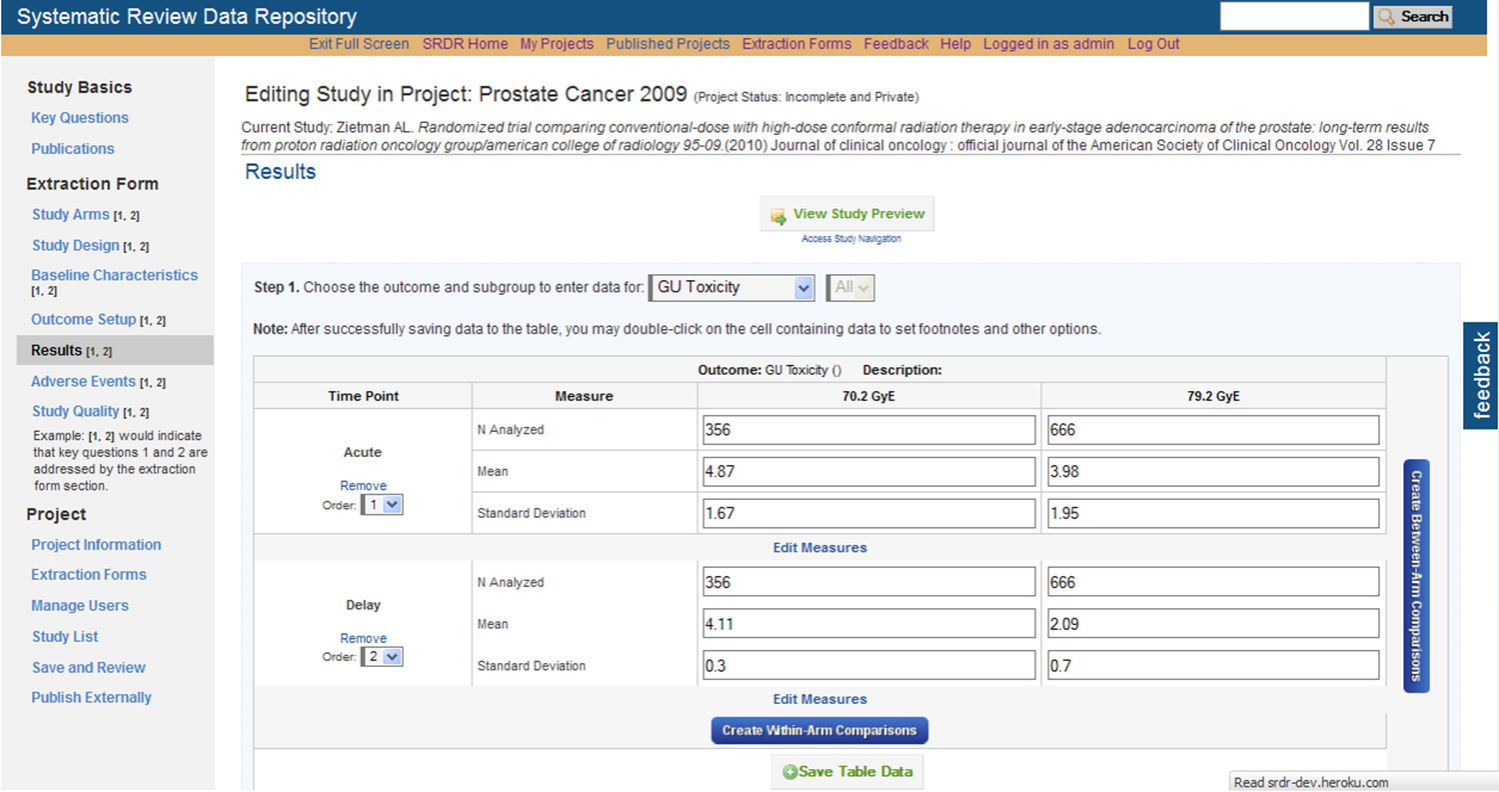
**Partial view of the Outcome Data Entry Section**.

**Figure 4:**
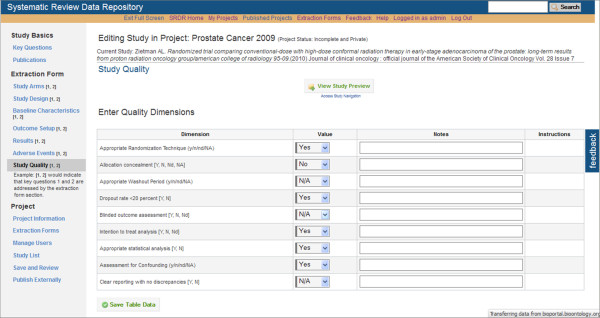
**Partial view of the Study Quality Appraisal Entry Section**.

**Figure 5:**
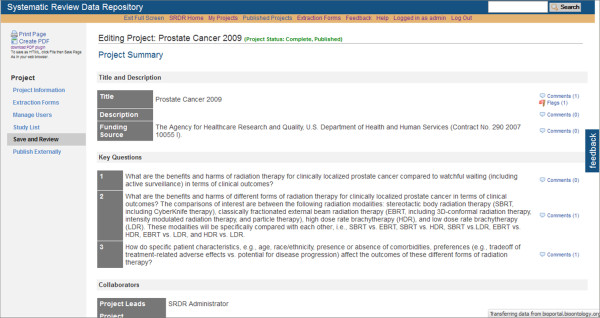
**Partial view of the Project Description and Key Questions Section**.

At this time, data extractors within the Tufts EPC have been testing SRDR functionality, yielding continuing improvements. Selected screenshots of the current implementation of the SRDR are depicted in Figures 1, 2, 3, 4 and 5. The focus of the archive is on enlisting future data collection, as we do not anticipate that many investigators will enter previously extracted data. However, we are creating import tools to simplify copying data extracted in other databases into the SRDR. This will enable those who wish to continue using their preferred software for data extraction to easily contribute previously extracted data into the SRDR archive.

Additional functionality currently under development prior to deployment includes a data comparison tool for use in double data extraction, a summary table creator and linking the SRDR to other databases (for example, ClinicalTrials.gov [3]). The next stage of deployment is scheduled for June 2012, when the SRDR will be opened to other AHRQ-funded EPCs, with general availability to be provided shortly thereafter.

### Next steps

Notably, there exist a number of registries or complementary resources with similar ethos, including the aforementioned International Clinical Trials Registry Platform [2] and ClinicalTrials.gov [3,6], the Human Studies Database [7,8], the Guidelines International Network International Guidelines Database [9-11] and the Centre for Reviews and Dissemination's international register of ongoing systematic reviews [12,13]. Interoperability among these organizations and resources would be of mutual benefit. Our intent is not to create a one-size-fits-all tool, or to supplant existing ones, but rather to improve, complement and extend existing research infrastructure. While several other commercial and professional ventures, such as Doctor Evidence [14] and the American Dietetic Association Evidence Analysis Library [15,16], have undertaken extracting data from published studies into dedicated databases, these endeavors remain proprietary and restricted resources. The SRDR is designed to be an open-access and freely available system. To our knowledge, this proposed archive is unique in its scope and mission, and will provide a tool sorely lacking in the research community.

Over the next phase of development, the Tufts EPC will continue to refine the SRDR and expand its features and functionality. We have formed a new expert panel to solicit further feedback as the repository becomes more widely used and to seek a greater range of stakeholder input. In addition to the relevant federal agencies, we have representatives from other EPCs, the Cochrane Collaboration, organizations that produce guidelines, as well as other relevant health care organizations, such as Kaiser-Permanente, to assist with further policy development. Many of the aforementioned organizations are working on databases for other kinds of information related to systematic reviews, including abstracts of systematic reviews and summaries of primary studies. It is our hope that by engaging these other organizations and the wider stakeholder community - patients, payers, researchers and governments alike - we can foster a collaborative environment that will enable this repository to grow into a valuable and permanent resource of benefit to all.

## Competing interests

The authors declare that they have no competing interests.

## Authors' contributions

JL conceived the idea of the SRDR. RI wrote the initial draft. SI, RI, NH, SK, CP, EB and JL edited and revised the final manuscript. All authors read and approved the final manuscript.
